# Evaluating the effects of geochemical and anthropogenic factors on the concentration and treatability of heavy metals in Awash River and Lake Beseka, Ethiopia: arsenic and molybdenum issues

**DOI:** 10.1007/s10661-023-11674-z

**Published:** 2023-09-12

**Authors:** Yosef Abebe, Paul Whitehead, Tena Alamirew, Li Jin, Esayas Alemayehu

**Affiliations:** 1 Africa Center of Excellence for Water Management, Water Science and Technology, AAU, Addis Ababa, Ethiopia; 2 Water and Land Resource Center, Ethiopian Institute of Water Resources, Addis Ababa University, Addis Ababa, Ethiopia; 3 Department of Ecohydrology and Water Quality, Ministry of Water and Energy, Addis Ababa, Ethiopia; 4 School of Geography and the Environment, University of Oxford, Oxford, OX1 3QY UK; 5 Geology Department, State University of New York at Cortland, Cortland, NY 13045 USA; 6 Faculty of Civil and Environmental Engineering, Jimma Institute of Technology, Jimma University, Jimma, Ethiopia

**Keywords:** Arsenic, Awash River, Heavy metals, Lake Beseka, Molybdenum

## Abstract

In the Awash River basin (Ethiopia), massive urbanization and industrialization, driven by rapid development and human settlement, are detrimental to the environment and human health as pollutants such as heavy metals (HMs) find their way into water bodies without proper treatment. The purpose of this study was to assess the HMs content and pollution sources within the basin. In this context, a total of 205 samples were collected from 21 surface water sampling stations. Heavy metal concentrations were measured using the Perkin Elmer NexION 350 ICP-MS with inductively coupled plasma. Findings demonstrate that high levels of HMs, such as Al, Mn, Mo, As, V, Fe, and Ba were exhibited with the value of 1257 μg/L, 626.8 μg/L, 116.7 μg/L, 61.2 μg/L, 100.5 μg/L, 1082.7 μg/L, and 211.7 μg/L, respectively. Among 20 HMs analyzed, 20% of the parameters within the study area were above the WHO limit for drinking water; Al (157 μg/L), V (100.5 μg/L), Fe (1082.7 μg/L), Mn (626.8 μg/L), and Mo (103.8 μg/L) were exhibited at sites along the river system. Likewise, 57% of water samples showed high values of As at many stations down the river systems. In particular, high HM concentrations seen in the upper Awash are primarily controlled by anthropogenic activities such as untreated industrial, agricultural, and domestic discharges, while the high HM concentrations in the middle Awash samples were likely due to the influence from the Lake Beseka that has high HM concentrations due to geological process. In conclusion, securing potable water for the rapidly increasing population in Addis Ababa and in the watersheds of Awash is unsafe to sustain the environment and the human health.

## Introduction

Wastes released from industry, domestic effluents, and agricultural runoffs have an impact on water bodies and are a subject of overgrowing concern worldwide. Heavy metals (metalloids) such as arsenic have severely contaminated the groundwater (GW) and surface water (SW) in Africa (Shaji et al., 2021). In addition to this, growing industrialization and urbanization (Das et al., 2009; Farkas et al., 2007; Wang et al., 2012) in the Awash River watershed has severely damage the ecosystem due to the toxin discharged into water bodies. Heavy metal (HMs) pollution in the SW has become a growing concern for the environment and people’s health (Ali & Khan, 2018; Huang et al., 2019; Jafari et al., 2018; Ravenscroft et al., 2009; Tiwari et al., 2015; Tiwari & Singh, 2014; Zhang et al., 2007). Pollutants come from a number of sources, primarily anthropogenic and geogenic (Bhuiyan et al., 2011; Muhammad et al., 2011; Ntengwe, 2006; Wei & Yang, 2010). When excessive amounts of pesticides and fertilizer are washed downstream by rain, they endanger aquatic life and have even been linked to the eutrophication of water hyacinth at Koka dam. Agricultural runoff may be a cause of HM pollution (As, Cd, Cu, Pb, U, and Zn) in aquatic bodies, and industrial disposal could also lead to high HMs such as As, Cd. Cr, Hg, Ni, Zn, and Pb concentrations.

Many countries have experienced similar problem, with anthropogenic sources like industrial wastewater such as mercury from Chlor-alkali plants, mining, and smelter wastes, such as arsenic and cadmium (Muibat et al., 2016), urban run-off (Alexandra et al., 2020), particularly lead, agricultural run-off, atmospheric deposition, and leaching from solid waste dumps (He et al., 2005; Kimbrough, 2009; Paul, Clement, et al., 2012) and natural sources (i.e, weathering of rocks, leaching of soils, volcanic ash). Anthropogenic activities like urban wastewater discharge contain a variety of pollutants including salts, metals, metalloids, pathogens, residual drugs, organic materials, and active residues of personal care products. The other potential sources relating to the water chemistry in a basin are the types of geologic materials that are present and the length of time that water is in contact with those materials. Natural activities in the form of oxide ores (Al, Mn, Sb) and sulfides ores (Fe, As, Pb, Zn, Cu, and Ni) and atmospheric deposition (As, Cd, Cr, Cu, Pb, Hg, and U) have shown the impact to the water chemistry from geological processes in the basin. For instance, in the middle valleys of the Awash Basin, the movement of water between GW and SW provides a major pathway for chemical transfer between terrestrial and aquatic systems (Tesedenya, 2018), which greatly affects the lake water qualitatively as well as quantitatively (Kalbus et al., 2006; Lamontagne et al., 2005; Schmidt & Schubert, 2007).

Industrial waste discharged into nearby bodies of water, such as rivers, raises water temperature and slows organism metabolism. This increases their need for oxygen. The smell of heavily polluted rivers like the Modjo, Akaki, and Atbella Rivers is intolerable. The discharge of effluent from wastewater treatment plants (WWTPs) has major detrimental effects on the health of aquatic ecosystems (Abebe et al., 2023; Yimer et al., 2020). Despite numerous studies showing that the concentration of pollutants, such as HMs, in streams has been rising and degrading the basin's water sources. There is still debate as to how much large- and small-scale agriculture has been practiced in the region (urban and peri-urban areas). However, almost all scales of agriculture are using river water for agricultural uses. In addition, the river Awash is also serving a source of domestic water supply (for Adma town and Methara town). Hence, depending on the chemical composition of the minerals that are weathered, the relative abundance of the major inorganic chemicals dissolved in the water changes, and the water chemistry of surface waters, including lakes and rivers, has been affected by the pollutants drained to them (Lewis et al., 2021) Once polluting substances are introduced into river systems, they are transported and transformed by physical, chemical, biological, and biochemical processes. Most likely, the widest spread of geogenic contamination affects human health significantly, with impacts such as elevated concentrations of As (Smith et al., 2000; Annette et al., 2008), non-specific health effects, causing cancer (Murgo, 2001), and also a detrimental impact on a human (Kim & Kim, 2015; Zeng et al., 2019).

As a result, the surface water bodies are becoming polluted, due to potential sources combined in a river system contaminating water resources and posing a serious water security problem in the basin (Esayas & Bernd, 2009). The effects of consuming HMs from various indirect sources such as in soil (Esmaeili et al., 2021; Sun et al., 2013), vegetables (Fathabad et al., 2018), food items (Fakhri et al., 2018), and food chains (Muchuweti et al., 2006) were detected as a result of polluted water-based agriculture. Exposure to these kinds of environmental contaminants thereby raises the likelihood of environmental and human health concerns (Fei et al., 2018). The effects of consuming HMs from various indirect sources such as in soil (Esmaeili et al., 2021; Sun et al., 2013), vegetables (Fathabad et al., 2018), food items (Fakhri et al., 2018), and food chains (Muchuweti et al., 2006) were detected as a result of polluted water-based agriculture. Exposure to these kinds of environmental contaminants thereby raises the likelihood of environmental and human health concerns (Fei et al., 2018). In order to understand the water quality (WQ) dynamics of the ARB and to ensure safe drinking water, sampling has been carried out by the Awash Basin Authorities, for the analysis of physicochemical issues (Yimer & Geberekidan, 2020; Yimer & Jin, 2020), nutrient problems including eutrophication (Bussi et al., 2021), and salinity (Jin et al., 2021). However, the HMs determination and associated concerns with human health and environmental impacts and their concentrations have not been studied extensively. Thus, the purpose of this study was to evaluate the spatial and temporal variations of HMs and their potential sources in the Awash basin using combined field sampling, laboratory analysis, and statistical analysis.

## **Materials and methods**

### Description of the study area

**Fig. 1:**
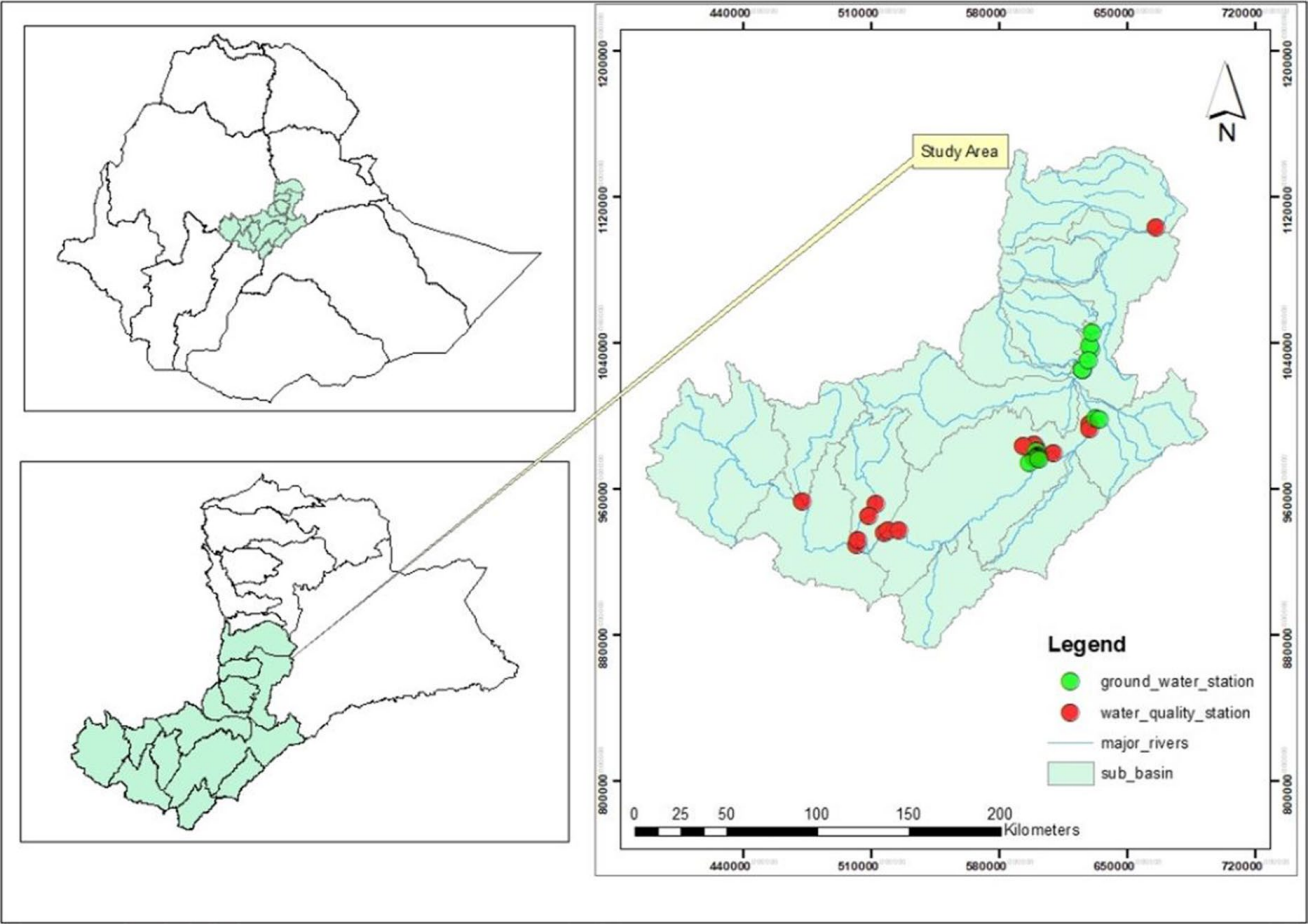
The map of the study area and sampling locations

The Awash Basin is the most developed, utilized, abused, impacted, and most populous (over 15% or nearly 18.6 million out of 120 million) basin in Ethiopia (Kebede et al., 2021). Awash River basin is the fourth and seventh in terms of area and volume of water, respectively. The basin covers a total area of 112,000 km^2^ and has an annual flow of 4.9 billion m^3^. More than half of the basin is covered by this study, which consists of the upper and middle valleys of the basin Awash (Fig. 1). The research area spans more than half of the basin and includes Awash 7 Kilo, Ambo, Sebeta, Bishoftu, Gelan, Adama, Modjo, and other towns, as well as the major city of the country Addis Abeba. It also contains more than 50% of the potential polluting industries. These include tanneries, paint factories, slaughterhouses, textiles, breweries, soft drink factories, sugar factories, hospitals, and pharmaceuticals. Additionally, the research area is vulnerable to water contamination, scarcity, and flooding.

### Study design Beseka

Water bodies, including tributaries, the Koka Dam, and Lake Beseka, as well as gauged and ungagged stations along the main Awash River basin, were sampled. Grab sampling methods were frequently used to take samples in the middle of the month. To reduce the risk of contamination, the plastic bottles were rinsed. Samples were collected on February 2020 and April, June, July, and October 2021. The sampling frequency was carried out twice a year for HM analysis (20 HMs) and four times a year for physicochemical analysis (some WQ parameters), depending on the spatial and temporal variability of the basin.

### Sampling stations/study area

In three sub basins of the Awash basin, sampling locations were chosen based on accessibility, pollution load, the presence of unsettling influences, the availability of a stable stream bed, safety, and security. For this task, a total of 125 physicochemical samples and 80 HMs samples were collected from 21 surface water and groundwater locations for 18 months.

### Sample collection procedures

The representative water sample was collected using polyethylene plastic bags and the prescribed procedures. As a result, a sample of water was obtained from 21 stations using grab sampling methods. Before collecting 1.5 l of water samples from the sample stations for the majority of physicochemical and heavy metals analyses, the sample containers were rinsed three times using distilled water. This was done to prevent any cross-contamination from previous samples. We left an airspace equivalent to about 1% of the container volume to account for thermal expansion during transport. Sample containers were sealed using self-adhesive paper that includes the sample ID, time, and date of collection, in order to detect unauthorized tampering with samples up until the time of analysis. Samples were acidified and air-freighted from Ethiopia to the Department of Earth Sciences at Oxford University, UK, and allowed to equilibrate overnight to permit re-dissolution of any precipitates or materials adsorbed to the bottles.

### Data quality management and analysis

Measurements of Mo, As, Sr, Ba, Fe, Cr, Al, Mn, U, V, and Zn were conducted by inductively coupled plasma mass spectrometry (ICP-MS) using a Perkin Elmer NexION 350D instrument, which was coupled with an Elemental Scientific prepFAST M5 autosampler (Table 1). For the study, the instrument was calibrated using the method of external calibration, where the concentration for the measured sample set was extrapolated from linear regressions generated from raw counts per second data from a series of standards. All blanks, standards, and samples were diluted using 2% v/v nitric acid (HNO_3_^−^) and doped Rn, In, Ir, and Re internal standards to normalize for any instrument drift. Additionally, for quality control purposes, an external standard was diluted and measured from a custom-bought-blended QMx multi-elemental standard to verify the calibrations. The certified reference materials (CRMs) SLRS-6 (river water standard) from NRC Canada and SPS-SW2 surface water standard LGC-UK were also measured in conjunction with the samples.

**Table 1:** Instrument operating conditions /ICP-MS setup parameters

Component/parameter	Type/value/mode
Nebulizer	PerklinElmer micromist
Spray chamber	Quartz cyclonic at ambient temperature (ca 2 °C)
Plasma gas flow	18 L/min
Nebulizer gas flow	0.9–1.0L/min
Sample uptake rate	250 μL/min
RF power	1600 W
Internal standards	Rn, In, Ir, and Re
Modes of operation	Helium collision cell and standard operation

### Laboratory analysis

In situ analysis was made to analyze pH, temperature, electrical conductivity (EC), and total dissolved solids (TDS) using a multi-meter and a partly portable Palintest micro 800 multi-meter, while alkalinity, bicarbonate, carbonate, and total hardness (TH) were measured using a 7500 photometer. At present, different techniques have been used for the determination of HMs, including AAS, XRF, ICP-MS/OES, and AAS (Al-Saydeh et al., 2017). In the study, ICP-MS was used to determine the concentration and types of HMs present in the SWs of the ARB.

### Statistical analysis

The Pearson correlation method was used to determine the correlation coefficient (*r*) between the variables. Analysis of SW samples was compared to WHO limits. The Turkey-Kramer test was used to compare the quality of SWs across all sites using an analysis of variance at a 5% significance level. Data analysis was made using SPSS version 23 and Minitab statistical package version, which were used for the day to identify the sources of actions and assess the commonalities; principal component analysis and factor analysis were utilized (Ashayeri et al., 2018; Yang et al., 2018).

## Results and discussions

### Physicochemical parameters (pH, EC, TDS, TH, and Alk) of surface water samples

The total dissolved solid (TDS) concentration proves a measure of dissolved inorganic chemicals present in the water samples. In the study, the highest TDS was observed in SW13 at 2482 mg/L in the dry season exceeding the threshold of WHO (1500 mg/L). This high value of TDS indicates high salinity and makes the SW of Lake Beseka (LB) less suitable for drinking and irrigation uses. Salinity is the cumulative sum of the cations and anions present in the water that has a significant impact on the soil salinity and degrades the palatability of the surface water. In fact, salinity does not cause serious health effects as compared to other geogenic contaminants, however, which is a potential indicator for the presence of dissolved ions in the water samples. A high TDS value was also observed in SW1. The pH ranged from 7.86 to 9.5. This high pH value (9.5) was observed at station SW13. The concentrations have good associations with GW pH, being greater under alkaline conditions, regardless of whether oxic or anoxic (Ayotte et al., 2011; Smedey & Kinniburgh, 2001). Similarly, the highest EC value (3650 μS/cm) was observed in SW13 (Table 2). It was above the maximum allowable limits of WHO. While the lowest EC value of 172 μS/cm was observed at SW6, in the wet season.

**Table 2:** Physicochemical parameters in the surface water sampling stations

Parameters	pH	Tur-NTU	EC-μS/cm	TDS-mg/L	Alk-mg/L	Bic.-mg/L	Car.-mg/L	TH-mg/L
Max	9.65	1050	3650	2482	2850	3500	1700	410
Min	6.59	1.52	172.4	86	40	45	25	15
Range	3.06	1048.5	3477.6	2396	2810	3455	1675	395
Mean	8.2	230.47	1019.16	589.34	525.43	639.07	315.21	127.46
St. Dev.	0.76	326.06	927.17	588.54	621.07	755.97	371.81	87.13
WHO, 2011	6.5–8.5	5 NTU	700 μS/cm	1000 mg/L	500 mg/L	580mg/L	250 mg/L	120 mg/L
FDRE-EPA	6–9	–	1000 μS/cm	–	–	–	–	–

N.B: (FDRE-EPA, 2003: Ethiopian Ambient Water Quality Standard (ETAWS). Not yet ratified)

### Physicochemical parameters (pH, EC, TDS, F, Cl, and Alk) of groundwater samples

Out of sixteen GW samples collected from the middle valley of Awash, seven samples were particularly selected to compute the quality of the GW and the SW interactions in the LB catchment. Accordingly, the findings show that high concentrations were exhibited with the mean and range values pH 8.74 (10.15 to 8.21), EC 3152 μS/cm (15645 μS/cm to 517 μS/cm), TDS 2099 mg/L (10826 mg/L to 268 mg/L), fluoride 7.85 mg/L (18.2 mg/L to 1.97 mg/L, chloride 244.54 mg/l (412.5 mg/L to 117.5mg/L), and alkalinity 885 mg/L (1600 mg/L to 225 mg/L) all are above the limits of WHO. For instance, high values of fluoride were seen in stations GW1 (18.2 mg/L) and GW2 (12.45 mg/L) and also in GW8 (13.5 mg/L), GW11 (15.55 mg/L), GW12 (12.15 mg/L), and GW13 (15.35 mg/L) were exhibited near LB (deep wells) and in Amibera irrigation sites (piezo stations), respectively. Previous studies indicate that the lake water chemistry has been mainly affected by the GW interaction or recharge due to GW to SW flow because; GW flux is the major component of the lake’s water input Dinka, 2020).

### Spatial and temporal variability of heavy metal concentration

**Fig. 2:**
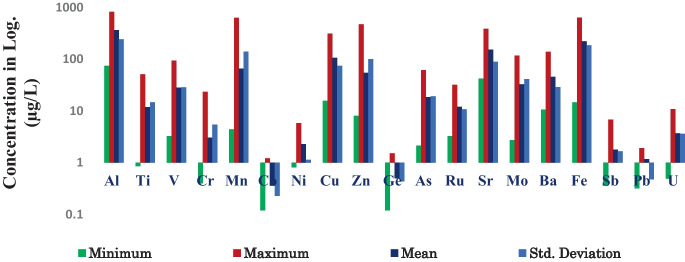
Spatial variability of heavy metals in statistical values

The spatial and temporal distribution of HMs and metalloids in the SWs was evaluated (Table 3). The range of these HMs (metalloids) in SWs was distributed evenly and found highly significant variability. For instance, the mean concentration of HMs increased as going from upstream catchments starting at station SW1 into downstream stations like SW21. The concentrations of these HMs (metalloids) (Ti, Cr, Co, Ni, Cu, Zn, Ge, Rb, Sr, Ba, Sb, Pb, and U) were within the WHO limits (Fig. 2), while the concentration of HMs (metalloids) like Mn, Mo, As, Al, V, and Fe in the sampled waters exceeded the WHO standards (WHO, 2011).

**Table 3:**
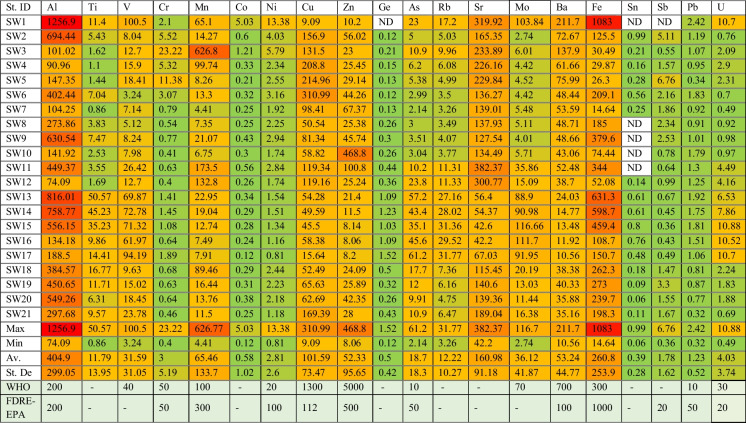
Spatial distribution of heavy metals in surface water (Awash River, Modjo River, and Lake Beseka) in Awash River Basin

NB: twenty-five percent of sampling stations, namely, SW13, SW14, SW15, SW16, are SW17 are located in LB and were selected from the LB, and showed high values of HMs Ti, Mo, Ge, As, Rb, V, and As, which might highly be associated with the rift tectonic natures. (FDRE-EPA, 2003: Ethiopian Ambient Water Quality Standard (ETAWS). Not yet ratified)

Bold entries illustrates the level of concentration of heavy metals from low concentaration (green color) to high concentaration (red color)

### Heavy metal concentration

Table 4 contrasts the findings from the same watersheds and those from the Awash River basin. Samples from the study showed higher average concentrations for most compared heavy metals, with the exception of some elements like Fe and Zn, according to a comparison between other basin-wide studies in the Awash River basin. The concentration of Cr, As, Ni, and Mo in this study is higher than the mean values in reports from previous studies, according to a comparison with data from previous studies.

**Table 4:** Correlation between variables/linear relation using SPSS version 23

Par.	Al	Ti	V	Cr	Mn	Co	Ni	Cu	Zn	Ge	As	Rb	Sr	Mo	Ba	Fe	Sn	Sb	Pb	U
Al	1																			
Ti	.638**	1																		
V	.348*	.579**	1																	
Cr	−.140	−.130	−.139	1																
Mn	−.165	−.139	−.071	.740**	1															
Co	.417**	−.016	.328*	.270	.365*	1														
Ni	.368*	−.143	.082	.406**	.526**	.914**	1													
Cu	−.151	−.150	−.304	.245	−.114	−.113	−.074	1												
Zn	−.082	−.202	−.279	−.115	−.061	−.055	−.019	−.087	1											
Ge	.297	.667**	.934**	−.202	−.057	−.093	−.316*	−.395*	−.220	1										
As	−.012	.288	.707**	−.114	.062	.002	−.111	−.404**	−.225	.787**	1									
Rb	.176	.555**	.889**	−.054	.124	.103	−.052	−.369*	−.269	.923**	.818**	1								
Sr	−.189	−.432**	−.133	.177	.475**	.339*	.387*	.012	.004	−.327*	−.080	−.126	1							
Mo	.212	.523**	.931**	−.190	−.083	.195	−.044	−.305	−.271	.898**	.741**	.932**	−.207	1						
Ba	.136	−.302	−.059	.490**	.649**	.781**	.928**	−.126	.031	−.407*	−.138	−.134	.541**	−.192	1					
Fe	.920**	.724**	.507**	−.176	−.127	.466**	.336*	−.277	−.108	.498**	.109	.331*	−.203	.387*	.100	1				
Sn	.459*	.228	.268	−.167	−.336	−.033	−.006	.352	−.239	.184	.111	.250	−.455*	.365	−.251	.221	1			
Sb	.042	−.282	−.318*	−.030	−.158	.014	.302	.263	−.042	−.441**	−.279	−.376*	.184	−.398*	.366*	−.221	.164	1		
Pb	.400*	.393*	.407*	−.014	−.038	.368*	.305	.364*	.215	.303	.132	.362*	−.192	.415**	.104	.422**	.619**	−.034	1	
U	.034	.285	.875**	−.099	.036	.223	.019	−.328*	−.292	.806**	.771**	.913**	.052	.929**	−.060	.208	.145	−.284	.283	1

N.B: ** correlation is significant at the 0.01 level (2-tailed)

*correlation is significant at the 0.05 level (2-tailed)

N.B: stations descriptions: SW1 (Awash River after Akaki mix), SW2 (Awash River @ Zeway Road at the bridge), SW3 (Modjo River downstream of factories), SW4 (Modjo River @ old bridge), SW5 (Modjo River @ Modjo Tannery), SW6 (Lake Koka @ Koka dam), SW7 (Awash River after Adama water treatment), SW8 (Awash River @ Wonji bridge), SW9 (Awash River before Lake Beseka mix), SW10 (Awash River after treatment Methara water supply), SW11 (Metahra sugar factories mils waste before entering AR), SW12 (Merti camp sewage discharge before entering AR), SW13 (Lake Beseka @ canal), SW14 (Lake Beseka @ intake), SW15 (Lake Beseka @ old road, car wash, right side), SW16 (Lake Beseka @ old road, left side), SW17 (Lake Beseka @ Lodge), SW18 (Awash River after Lake Beseka mix), SW19 (Awash River @ Awash 7 water supply), SW20 (Awash River @ Weir site), and SW21 (Awash River @ Meteka bridge)

Bold entries implies a strong positive correlation between elements and the level of siginificance

#### Aluminum (Al), iron (Fe), and manganese (Mn)

**Fig. 3:**
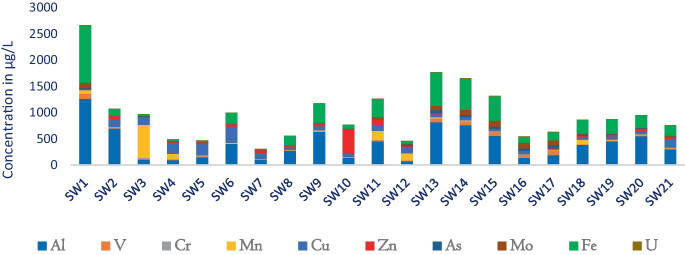
Heavy metals concentrations /Al, V, Cr, Mn, Cu, Mn, Zn, As, Mo, Fe, and U/

Naturally, the concentration of iron (Fe) in SW bodies is high. However, exceptionally the greatest portion of Fe above the recommended guideline values was exhibited in stations SW1, SW13, SW14, SW15, SW11, and SW9 with high values of 1082.7 μg/L (> threefolds compared to the allowable limit 10 μg/L), 631.3 μg/L, 598.7 μg/L, 459.4 μg/L, 379.6 μg/L, and 343.9 μg/L, respectively. Correspondingly, high concentrations of Mn varied from 626.8 μg/L to 4.41 μg/L were recorded. Manganese (Mn) exceeded the respective threshold values (100 μg/L) in SW3 (at Modjo River after industrial/tanneries wastes) 626.8 μg/L. Similarly, high values of Mn in stations SW11 (at Methara sugar factory mill waste) and SW12 (at Merti camp sewage discharge) exhibited 173.5 μg/L and 132.8 μg/L, respectively. The sources of pollution are mainly associated with industrial waste (SW3), sugar factories’ mill wastes (SW11), and sewage discharge (SW12) (Fig. 3).

In the study, the most common pollutants, namely, Zn, Cr, Co, Hg, As, Cu, Ni, Pb, and others like Fe, Mn, V, U, Sr, Sn, and Al, were analyzed. For instance, the highest concentrations of Al above the WHO limits were exhibited at stations SW1 (> sixfolds), SW13 (> fourfolds), SW14 (> fourfolds), SW9 (> threefolds), SW15 (> two and a half folds), SW20 (> two and a half folds), SW19 (> twofolds), SW11 (> twofolds), SW6 (> twofolds), SW8, and SW21 with the values of 1257 μg/L, 816 μg/L, 758.8 μg/L, 630.5 μg/L, 556.2 μg/L, 549.2 μg/L, 450.7 μg/L, 449.4 μg/L, 402.2 μg/L, 297.7 μg/L, and 273.9 μg/L, respectively. It implies that over 50% of water samples showed above the WHO limits (WHO, 2011). Perhaps, this spatial variability in stations SW1, SW6, SW8, SW9, and SW11 might come from industrial sources, in stations SW13, SW14, SW15, SW18, and SW19 natural sources (or leaching of soils, geochemistry), whereas stations SW18, SW19, SW20, and SW21 might be associated with the flux discharge come from industrial sources from LB.

**Fig. 4:**
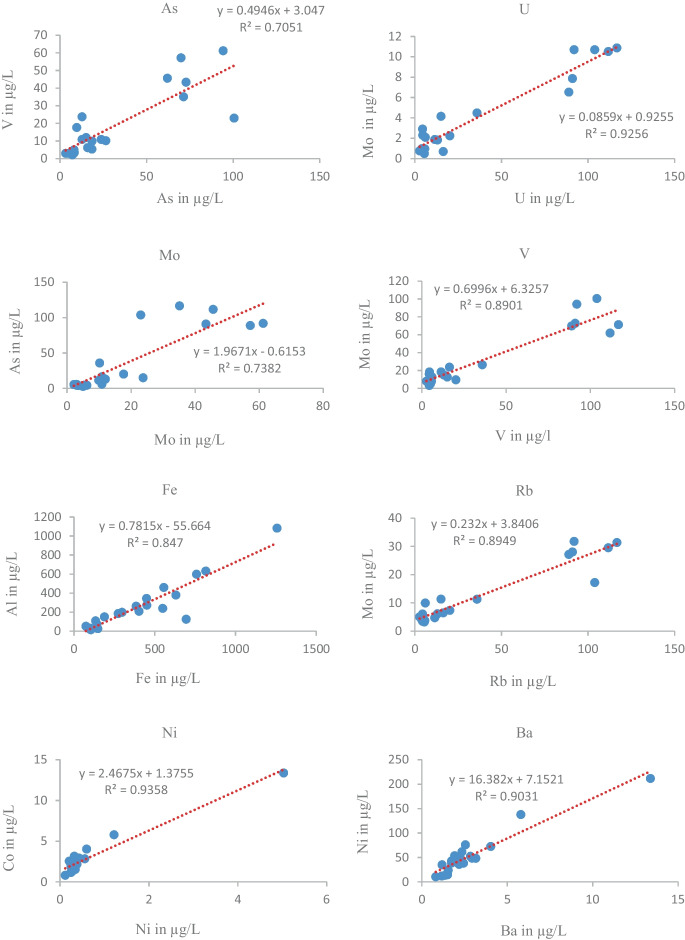
The trend line of some heavy metals

Due to natural process, localized enrichments of some elements, namely, Al, Mn, Cu, Zn, Sr, and Fe, were found with a value of 449.4 μg/L, 173.5 μg/L, 119 μg/L, 100 μg/L, 382 μg/L, and 343.95 μg/L, respectively. In the study, samples that impacted HMs pollution were mainly from industrial wastes derived (upstream Koka) and rock weathering (GW-SW) interactions. Titanium (Ti) ranged from 50.57 to 0.86 μg/L with mean values of 11.8 μg/L. A high concentration of Ni was observed in SW1 and SW17 with a value of 13.38 μg/L and 0.81 μg/L, respectively, with a mean value of 2.81 μg/L. Cobalt (Co) varied 5.03 μg/L and 0.12 μg/L in stations SW1 and SW17 respectively. The assessment of Ba exhibited high concentration in SW1, ranging from 211.7 to 10.56 μg/L (SW17) with a mean value of 53.24 μg/L. The concentration of HMs, namely, Ti, Co, Ni, Ge, Rb, Sr, Ba, Sn, Sb, and Pb, exhibited below the limits. The concentration of Ti was high in stations SW13, SW14, SW15, SW16, and SW17 due to natural activities. It varied from 50.57 μg/L (SW13) to 0.86 μg/L (SW7) with a mean value of 11.79 μg/L (Fig. 4). The amount of Ge ranged from 1.52 μg/L (SW17) and 0.12 μg/L (SW2) with a mean value of 0.50 μg/L. A high Rb concentration was exhibited in station SW17 (31.77 μg/L) and a low concentration in SW7 (3.26 μg/L) with a mean value of 12.2 μg/L.

#### Vanadium (V) and uranium (U)

The uneven distribution of HMs such as V and U tends to increase slightly downstream of LB. The concentrations of V and U varied from 100.5 to 3.24 μg/L and 10.88 μg/L and 0.49 μg/L, respectively. High concentrations of V and U were exhibited in station SW1 due to industrial and urban wastes from Addis Ababa. Both V and U were high in LB, due to the features of the lake catchment, the aquifer, and volcanic ash. In the study, particularly in these two areas, high levels of HMs have been identified. Most of the heavily concentrated area is LB due to rock weathering.

#### Tin (Sn), antimony (Sb), lead (Pb), copper (Cu), nickel (Ni), and barium (Ba)

**Fig. 5:**
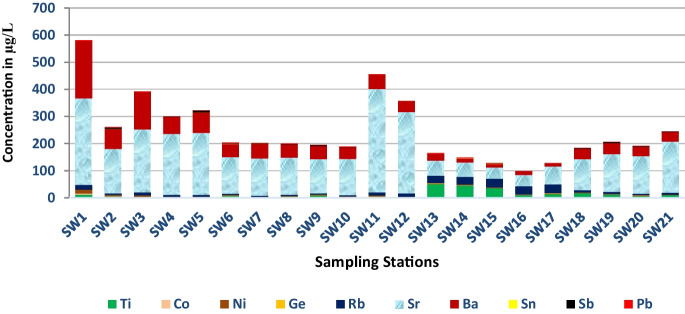
Heavy metals concentrations /Ti, Co, Ni, Ge, Rb, Sr, Ba, Sn, Sb, and Pb/

All samples of Sn, Sb, and Pb did not exceed the WHO. The concentration of Sn ranges from 0.99 μg/L to not detected (in SW1, SW8, SW9, SW10, and SW11) and with a mean value of 0.39 μg/L. The concentration of antimony (Sb) varies from 6.76 μg/L to not detected significant value in station SW5 and had a mean value of 1.78 μg/L. The concentrations of Pb in many of the samples were lower than the WHO permitted limit, ranged 2.42 μg/L (SW1) and 0.32 μg/L (SW21) with a mean value of 1.23 μg/L. The sources for the high concentration of Pb, Sn, Co, Ni, and Ba in stations SW1, SW2, SW1, SW1, and SW1, respectively, are anthropogenic sources, including industrial wastes, domestic wastes, sewage, and agricultural runoff. As shown in Fig. 5i, ii, contour plots were used to explore the relationships between variables.

#### Arsenic (As) and molybdenum (Mo)

In natural waters, As is mostly found in its inorganic form as oxyanions of arsenite As(III) or arsenate As(V). However, both forms are highly toxic inorganic species (Fendorf et al., 2010). In the study, the findings in Table 3 exhibited that the highest values of As above the limit of WHO (10 μg/L) were recorded in 57 % of water samples collected from stations SW1 (23.01 μg/L), SW3 (10.9 μg/L), SW11 (10.18 μg/L), SW12 (23.75 μg/L), SW13 (57.22 μg/L), SW14 (43.37 μg/L), SW15 (35.12 μg/L), SW16 (46.62 μg/L), SW17 (61.23 μg/L), SW18 (17.67 μg/L), SW19 (11.98 μg/L), and SW21 (10.89 μg/L). This heterogeneous spatial distribution of As in SW of ARB mainly derives from natural activities in the middle valley of Awash. The geogenic or volcanic ash contamination may be the cause for the elevated concentration of As in LB (Alcaine et al., 2020; Frascoli & Hudson-Edwards, 2018; Smedey & Kinniburgh, 2001).

The sources might also be the leaching of soil, volcanic ash, and rock weathering in the lake catchment and river waters, where As(V) is the dominant species. Therefore, the main sources for the presence of a high concentration of As in the SW of the ARB might be anthropogenic in SW1, SW3, and SW11. While the sources of As in stations SW12, SW13, SW14, SW15, SW16, and SW17 were predominantly geogenic; tectonic, clay, volcanic ash, and sand weathering phenomenon. Shockingly, high concentrations of As in stations SW18, SW19, and SW21 in the LB area were associated with the lake-river interaction and the discharge of the lake water flux into the ARB. Rhyolitic and volcanic ash in the Ethiopian Rift Valley (WHO, 2008), being a possible natural contaminant of GW sources (ARSLAND, 2006), elevate the concentration of As(V), which is predominantly accompanied by high V, Mo, and U concentrations (Alcaine et al., 2020; Smedley & David, 2017). Arsenic (As) is considered by some researchers to have more serious health effects than any other environmental contaminant (Smith & Steinmaus, 2009).

Molybdenum (Mo) occurs naturally in minerals, rocks, and soils as well as in aqueous form (with oxidation states of IV and VI) (Paullin et al., 2001). This study found that SW had a high concentration of Mo. Some, though not all, have intermixed felsic volcanic ash, which potentially contributes to an enriched and labile Mo source in Ethiopia. The highest amounts of Mo were recorded in six different stations; except SW1, the other five stations are from LB including SW13, SW14, SW15, SW16, and SW17 with the content of 103 μg/L, 88.9 μg/L, 90.98 μg/L, 116.7 μg/L, 111.7 μg/L, and 91.95 μg/L, respectively. The potential source of Mo for SW1 might be anthropogenic and industrial waste. While the remaining five stations are mainly associated with the geogenic natured of the studied water or/and the relative enrichment. Its source of pollution is totally geogenic, weathering, leaching, and volcanic eruptions have been reported (Paul, Clements, et al., 2012).

The lake waters have correspondingly high concentrations of U and V, F, and Mo 90 (Klemperer & Cash, 2007; Smedley et al., 2017). A large range of Mo concentrations was seen in lake waters, depending on ambient redox, pH, and salinity variations (Reimann et al., 2002). The range of Mo was found to be from 1.30 to 108.69 μg/L, which can be toxic in high doses. The highest values of Mo 116.7 μg/L (>70 μg/L) and As 61.2 μg/L (sixfolds greater than the allowable limits of WHO (>10 μg/L), were obtained in LB. Therefore, the measured highest concentrations of Mo and As at LBs stations might be due to the inflow of water (interaction of surface and subsurface). Surprisingly, unexpected levels of Mo, As, V, and U were observed in the same sites (SW1, SW13, SW14, SW15, SW16, and SW17). Of these six stations, five of them are from LB. Earlier, the highest values were exhibited by studies, with the mean value of Mo (246 μg/L) and As (41.2 μg/L) (Klemperer & Cash, 2007), Mo 250 μg/L, and As 67.3 μg/L were recorded in LB (Smedley et al., 2017). Thus, this situation boosts the mobility of Mo and which has some notable overlap with that of a number of other anions/oxyanions (Smedey & Kinniburgh, 2001; Smedley & Kinniburgh, 2002). Notably, Mo, U, V, and As revealed similar spatial and temporal homogeneity. The feasibility of Mo, and correlated with U, V, and As, with a significant Pearson correlation of 93% (*r* = 0.929), 93% (*r* =0.93), and 74% (*r* =0.741) respectively.

### Principal component and factor analysis

**Fig. 6:**
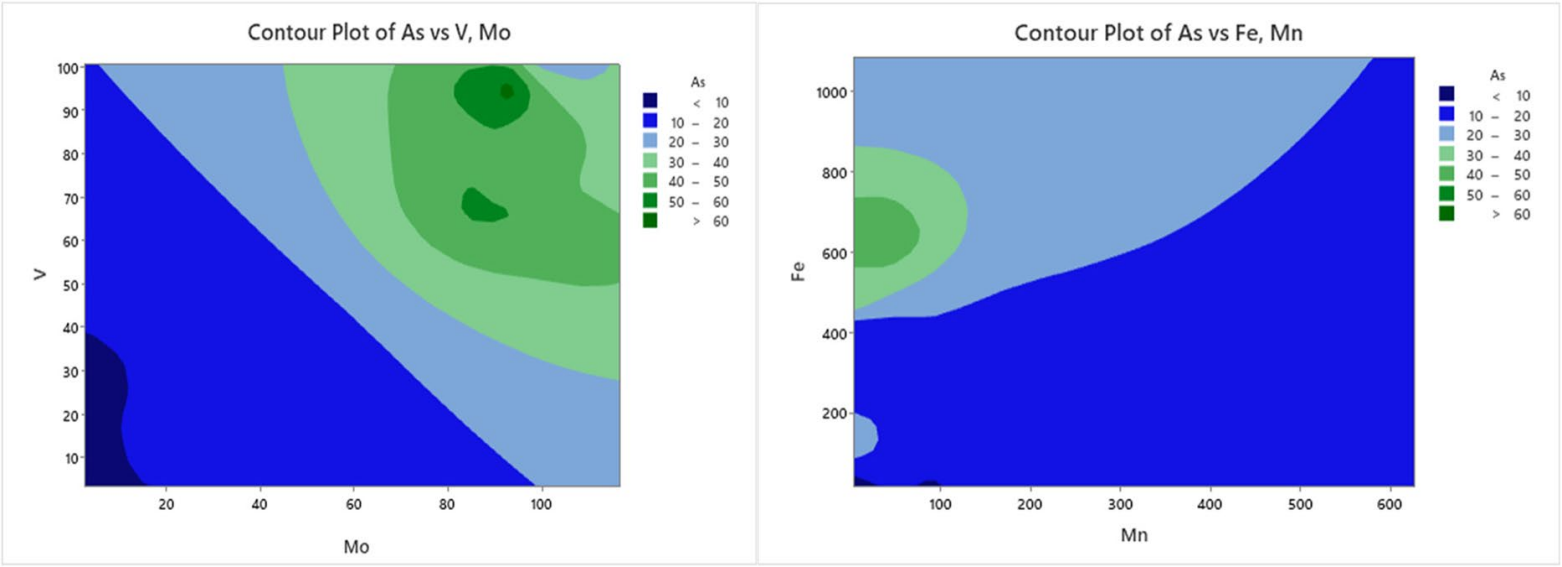
Contour plot of arsenic (As) with Mn, Fe, V, and Mo

As seen in Fig. 6, there was no point above the reference line, so this implies that the data analyzed did not significantly affect the analysis. The rotated factor loading analysis in factor one shows that high loading values of U, Mo, V, As, Ti, and Rb, had high influence values of 0.98, 0.97, 0.95, 0.918, 0.896, 0.549, and 0.525, and how large positive loading factor exhibited in factor one respectively. So, this factor indicates similar sources, which might be the natural activities (weathering of sedimentary rocks) and might leathering soils. In factor two, Ti had a small positive loading value of 0.364 and had a weak influence on the variables. Factor four, Cr, Mn, and Ba showed positively large loading factors with 0.93, 0.73, and 0.344 respectively. In sum, the loading results of the factors, and together all factors explained 0.804 or 80.4% of the variation of data.

### Source apportionment of heavy metals

#### Sources of heavy metal pollution before Lake Beseka mix (BLBM)

**Fig. 7:**
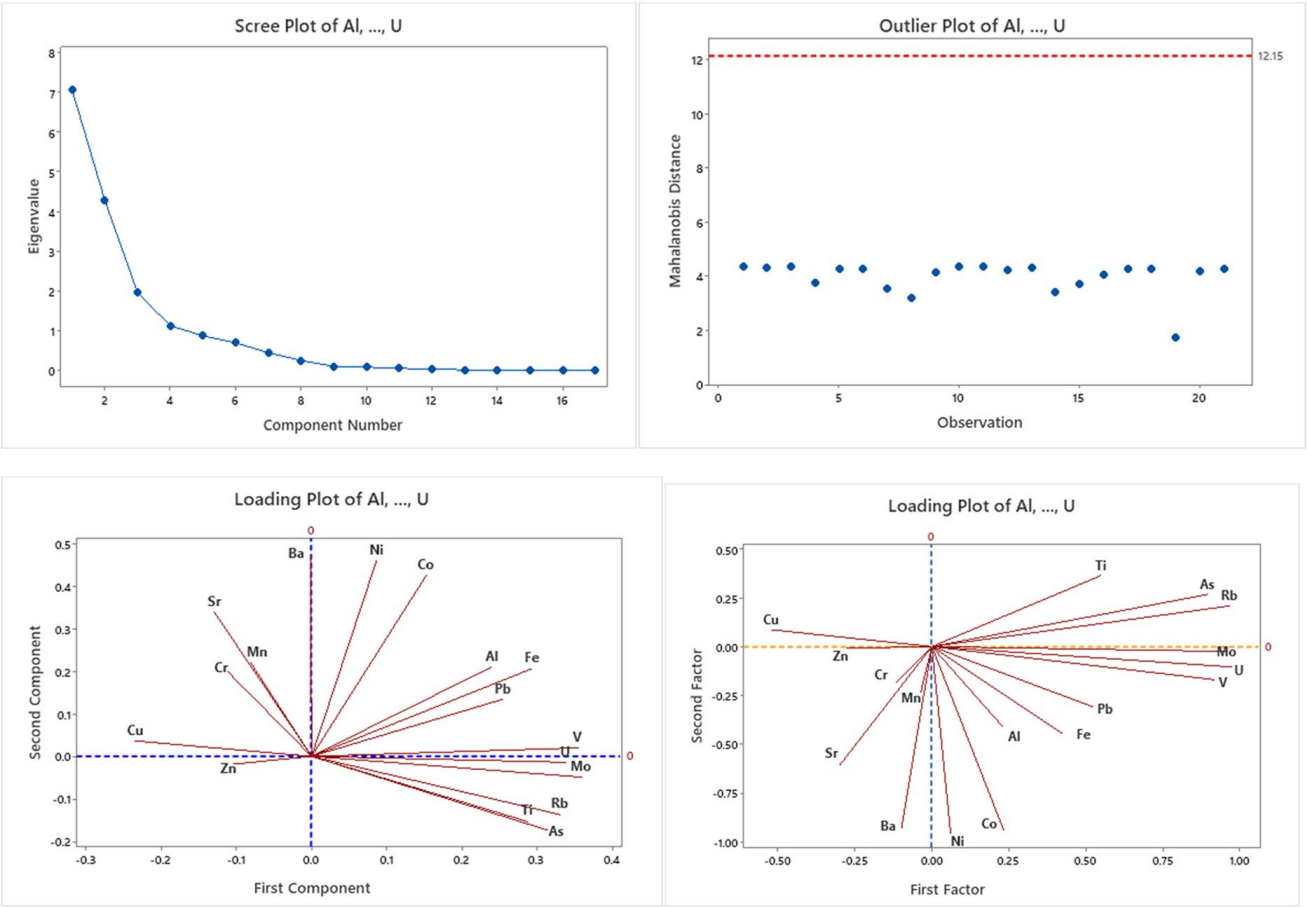
Screen plot, outlier plot, and loading plot using component and factor analysis

In the study, among 20 HMs analyzed, 20% of the parameters within the catchments (study area) were above the WHO limit, i.e., Al (1257 μg/L), V (100.5 μg/L), Fe (1082.7 μg/L), Mn (626.8 μg/L) and Mo (103.8 μg/L) at station SW1. Likewise, the highest values (even under the limit) of Cr (23.22μg/L), Co (5.03 μg/L), Ni (13.4 μg/L), Cu (310.99 μg/L), and Ba (211.7 μg/L) were recorded in stations SW3, SW1, SW1, and SW6, respectively (Fig. 7). Arguably, the main sources of pollution in the upper Awash River basin come from industrial and urban wastes, agricultural runoff (pesticides, fertilizers), and sewage discharge. The untreated urban and industrial wastes and agricultural runoff potentially contribute as primary sources and are also responsible for the incidence of HMs in the upstream Koka. Therefore, human activities and poor wastewater management contributed to the increasing concentrations of pollutants and resulted in the deterioration of the receiver water.

#### Sources of heavy metal pollution at Lake Beseka (LB)

Pollution sources in Lake Beseka water are mainly related to the geological properties of lake water as a fundamental source. The geology, geochemistry, and rift features of the lake basin may relate primarily to volcanic ash, a potential source of HMs in groundwater. As shown in Fig. 6, the water samples analyzed show high concentrations of HMs (metalloids). The values displayed on these stations exceeded WHO limits such as As, V, Mo, and U. Seasonal variations in HMs in lake water are directly related to variations in GW concentrations. It may be present due to the subsurface and groundwater interaction with the lake water; hereby, the sources of HMs concentration mainly come from the catchment geology and volcano cases. Accordingly, high concentrations of HMs such as V, As, and Mo exhibited in stations SW13, SW14, SW15, SW16, and SW17 with a concentration of 69.87 μg/L, 57.2 μg/L, and 62.03 μg/L; 72.78 μg/L, 43.4 μg/L, and 90.98 μg/L; 71.72 μg/L, 35.1 μg/L, and 116.66 μg/L; 61.97 μg/L, 45.6 μg/L, and 111.7 μg/L; and 94.19 μg/L, 61.2 μg/L, and 91.95 μg/L, respectively.

#### Correlation and regression between heavy metals

As shown in Table 3, ANOVA was performed, by using SPSS (Version 23) at a 95% confidence interval to determine the differences among the samples. The correlational analysis including Pearson’s correlation is an important basis for determining the sources of HMs. So, it was used to evaluate the correlation matrix between HMs (metalloids) in the SW within the basin. Table 3 illustrates a strong positive statistical correlation between Ge vs V, Ge vs Rb, Rb vs U, Ba vs Ni, and, Fe vs Al, with correlation coefficients of 0.934 (93.4%), 0.923 (92.3%), 0.913 (91.3%), 0.928 (92.8%), and 0.92 (92.0%), respectively.

**Fig. 8:**
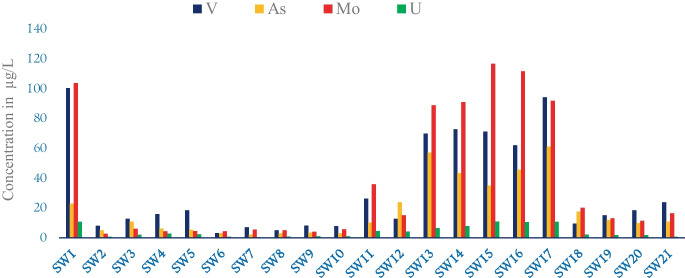
The concentration of heavy metals /Mo, As, U, and V/

Similarly, the significant spatial differences (*ρ* < 0.05) between Ge and U, As and Rb, As and Ge, Ba and Co, As and U, Ti and Fe, and As and V were positively correlated with 80.6%, 81.8%, 78.7%, 78 %, 77%, 72.4%, and 70.7%, respectively. The proportion of variation was computed using *R* squared (*R*^2^) (Fig. 4). The proportion of variation was also computed using R squared (R^2^) (Fig. 8a–h). The figures prove that Al and Fe, As and Mo, As and V, Mo and V, Mo and Rb, Mo and U, Co and Ni, Ni and Ba, and account for 84.7%, 73.8%, 70.5%, 89%, 92.6%, 89.5%, 93.6%, and 90.3% of the variation respectively. Therefore, a plot of HMs (Fig. 8a–h) showed a strong linear fit, and the data obtained (*R*^2^) coefficients between the aforementioned parameters have a common origin (Yuanan et al., 2013).

#### Sources of heavy metal pollution after Lake Beseka mix (ALBM)

In the potential pollution sources of HMs (E.g As, Mo, V, and U) after Lake Beseka mix (ALBM), in stations SW18, SW19, SW20, and SW21, the source of pollution comes from the discharge of LB and the wastes discharged from sugar factory mills and Merti camp’s sewage discharge. Even though, the SW11 and SW12 and also the upstream discharge have an effect on it. Perhaps, the geogenic activities surrounding LB pollute the river water downstream of Methara. Already many years ago, the lake connected with the river water through an artificially constructed channel and enabled a dynamic exchange of underground materials (Furi, 2011). Degradation of LB SW chemistry due to GW inflow is becoming an urgent issue. This could be a possible source of high HM concentrations at stations SW13, SW1, SW15, SW16, and SW17. Although sources such as municipal waste (SW11), sugar factory waste (SW12), geogenic sources (SW13-SW17), and anthropogenic discharges can pollute the river at stations SW1, SW2, SW3, SW4, SW5, and SW6, in sum, due to daily activities, untreated sewage from industry, households, and municipalities has degraded water resources. Interactions between groundwater and surface lakes in Ethiopia’s Main Rift Valley and possible HM contamination in lake water samples showed the possibility of HMs increasing in lake water. However, due to the sparse data in Ethiopia’s Awash Basin HMs levels were not well studied on LB. Therefore, this study was conducted to evaluate the interaction between GW-SW and HM occurrence via groundwater interaction across the LB and AR.

## Conclusion

Spatial variability and an uneven distribution of HMs were observed in the Awash River basin, particularly in the upstream catchment (SW1, SW2, SW3, and SW4) due to industrial wastewater discharge while stations like SW13, SW14, SW15, SW16, and SW17 were highly polluted by trace metals discharged from industries and municipal waste. In the Lake Beseka region, the Ethiopian Rift Valley may have contributed to elevated HMs concentration including Mo, V, U, and As and surface–groundwater interactions brings high level of Fe, Mn, Cr, Sr, Al, Ba, and Zn from the groundwater to the lake water and river water in the central basin of the valley. In general, the WQ of the basin depends on many factors, including the proportion of surface runoff (industrial and domestic runoff, agricultural runoff, and sewage), rock erosion, and solid waste runoff from inland water bodies processes and mixing of incoming water of different quality and entry of pollutants and unwanted substances.

- High HM concentrations, such as Mo and As contamination in the central Awash Basin, were observed mainly due to geogenic activity, while Cr, Sr, Al, Ba, Fe, Mn, and Zn concentrations were particularly high in correlation at Akaki, runoff from anthropogenic sources and mostly lead in the upper reaches of the Koka.
- At the same time, high concentrations of As, Mo, U, and V were observed in LB (geogenic source) and also in upper Koka (due to anthropogenic and/or geogenic sources). Due to the increased risk of certain trace metals, relevant authorities in the study area, such as the Ministry of Water and Energy (MoWE), environmental protection authority (EPA), and Awash Basin Administration Office (AwBAO), must regularly monitor and investigate certain risk areas (LB and after-lake mixing).
- To reduce metal toxicity in AwRB, the Ethiopian government should improve wastewater management nationally. In addition, there is a need to ensure and protect the WQ and sustainability of waterways, and more regional studies on WQ in the basin are needed.

Overall, the study highlighted some new areas of water quality challenges. Both anthropogenic and geogenic activities contribute to the WQ degradation. Some of the new observations, however, need to be further investigated to arrive at the root causes of the problem and means to prevent further contamination of the river course.

## Data Availability

The data used and analyzed during this study are available if needed for further review

## Declarations

All authors have read, understood, and have complied as applicable with the statement on “Ethical responsibilities of Authors” as found in the Instructions for Authors and are aware that with minor exceptions, no changes can be made to authorship once the paper is submitted.

### Disclaimer

The views expressed and information contained in it are not necessarily those of or endorsed by FCDO, which can accept no responsibility for such views or information or for any reliance placed on them.
